# *N*′-[(1*E*,2*E*)-3,7-Dimethyl­octa-2,6-dien-1-yl­idene]pyridine-4-carbohydrazide

**DOI:** 10.1107/S1600536812009075

**Published:** 2012-03-24

**Authors:** Mashooq A. Bhat, Hatem A. Abdel-Aziz, Hazem A. Ghabbour, Madhukar Hemamalini, Hoong-Kun Fun

**Affiliations:** a Department of Pharmaceutical Chemistry, College of Pharmacy, King Saud University, PO Box 2457, Riyadh 11451, Saudi Arabia; b X-ray Crystallography Unit, School of Physics, Universiti Sains Malaysia, 11800 USM, Penang, Malaysia

## Abstract

In the title compound, C_16_H_21_N_3_O, the mol­ecule adopts an *E* conformation about the central C=N double bond. The 2-methyl­pent-2-ene group is disordered over two sets of sites, with a refined occupancy ratio of 0.785 (8):0.215 (8). The dihedral angle between the essentially planar [the r.m.s. value for the major component is 0.021 (7) and its maximum deviation is 0.025 (4) Å; the r.m.s. value for the minor component is 0.03 (4) and its maximum deviation is 0.05 (3) Å] major and minor components of the 2-methyl­but-2-ene group is 35.9 (13)°. In the crystal, C—H⋯O and N—H⋯O hydrogen bonds link the molecules, with the same O atom acting as the acceptor. This results in *C* ^1^_1_(4) and *C* ^1^_1_(5) [001] chains.

## Related literature

For details and the biological activity of isoniazide, see: Janin (2007 ▶); Maccari *et al.* (2005 ▶); Slayden & Barry (2000 ▶); Hearn *et al.* (2009 ▶); Tripathi *et al.* (2011 ▶). For related structures, see: Naveenkumar *et al.* (2010) ▶; Jiang *et al.* (2009 ▶); Khan *et al.* (2009 ▶). For hydrogen-bond motifs, see: Bernstein *et al.* (1995 ▶).
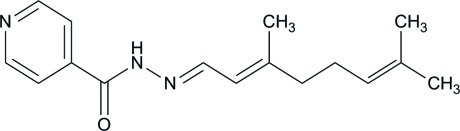


## Experimental

### 

#### Crystal data

C_16_H_21_N_3_O
*M* *_r_* = 271.36
Monoclinic, 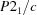

*a* = 17.5415 (8) Å
*b* = 12.0708 (6) Å
*c* = 7.8430 (4) Å
β = 101.854 (3)°
*V* = 1625.26 (14) Å^3^
*Z* = 4
Cu *K*α radiation
μ = 0.56 mm^−1^
*T* = 296 K
0.90 × 0.27 × 0.17 mm

#### Data collection

Bruker SMART APEXII CCD area-detector diffractometer
Absorption correction: multi-scan (*SADABS*; Bruker, 2009 ▶) *T*_min_ = 0.633, *T*_max_ = 0.912
16548 measured reflections
2978 independent reflections
2376 reflections with *I* > 2σ(*I*)
*R*_int_ = 0.027

#### Refinement

*R*[*F* ^2^ > 2σ(*F* ^2^)] = 0.049
*wR*(*F* ^2^) = 0.154
*S* = 1.03
2978 reflections
239 parameters
12 restraints
H atoms treated by a mixture of independent and constrained refinement
Δρ_max_ = 0.13 e Å^−3^
Δρ_min_ = −0.14 e Å^−3^

### 

Data collection: *APEX2* (Bruker, 2009 ▶); cell refinement: *SAINT* (Bruker, 2009 ▶); data reduction: *SAINT*; program(s) used to solve structure: *SHELXTL* (Sheldrick, 2008 ▶); program(s) used to refine structure: *SHELXTL*; molecular graphics: *SHELXTL*; software used to prepare material for publication: *SHELXTL* and *PLATON* (Spek, 2009 ▶).

## Supplementary Material

Crystal structure: contains datablock(s) global, I. DOI: 10.1107/S1600536812009075/lh5421sup1.cif

Structure factors: contains datablock(s) I. DOI: 10.1107/S1600536812009075/lh5421Isup2.hkl

Supplementary material file. DOI: 10.1107/S1600536812009075/lh5421Isup3.cml

Additional supplementary materials:  crystallographic information; 3D view; checkCIF report

## Figures and Tables

**Table 1:** Hydrogen-bond geometry (Å, °)

*D*—H⋯*A*	*D*—H	H⋯*A*	*D*⋯*A*	*D*—H⋯*A*
N3—H1*N*3⋯O1^i^	0.873 (17)	2.052 (17)	2.9167 (18)	170.8 (16)
C4—H4*A*⋯O1^i^	0.93	2.53	3.251 (2)	135

Symmetry code: (i) 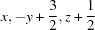.

**Fig. 1.**
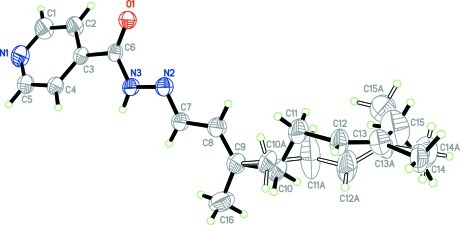
The asymmetric unit of the title compound, showing 30% probability displacement ellipsoids. Open bonds represent disordered components.

**Fig. 2.**
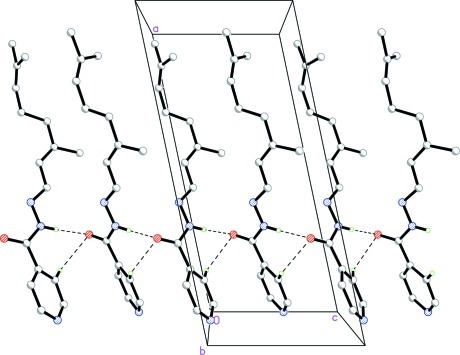
The crystal packing of the title compound (I). Hydrogen bonds are shown as dashed lines. The disorder is not shown.

## supplementary crystallographic information

### Comment

In the search of new compounds, isoniazid derivatives have been found to possess potential tuberculostatic activity (Janin, 2007; Maccari *et al.*, 2005; Slayden & Barry, 2000; Hearn *et al.*, 2009; Tripathi *et al.*, 2011). The crystal structures of (*E*)-N'- (2-Benzyloxybenzylidene)isonicotinohydrazide methanol solvate monohydrate (Naveenkumar *et al.*, 2010), *N*'-(1-Phenylethylidene)isonicotino hydrazide (Jiang *et al.*, 2009) and *N*'-(4-Bromophenylsulfonyl) isonicotinohydrazide (Khan *et al.*, 2009) have been reported in the literature. Here, we present the crystal structure of the title compound, (I).

The asymmetric unit of the title compound is shown in Fig. 1. The molecule adopts an *E* configuration about the central C7═N2 double bond. The 2-methylpent-2-ene group is disordered over two sets of sites, with a refined occupancy ratio of 0.785 (8):0.215 (8). The dihedral angles between the major and minor components of the 2-methylbut-2-ene (C11–C15:C11A–C15A) group is 35.9 (13)°.

In the crystal, Fig. 2, the adjacent molecules are connected *via* bifurcated N—H···O and C—H···O hydrogen bonds (Table 1), generating *R*^1^_2_(7) ring motifs (Bernstein *et al.*, 1995), resulting in supramolecular [001] chains.

### Experimental

The title compound was prepared by the reaction of citral, 3,7-dimethylocta- 2,6-dienal (0.15 g, 1 mmol) with isoniazid (0.14 g, 1 mmol) in ETOH/H_2_O (3:1, v/v, 10 mL). After stirring for 3 h at room temperature, the resulting mixture was concentrated under reduced pressure. The residue washed with cold ethyl alcohol and then with ethyl ether to afford the title compound. Colorless blocks of the latter compound suitable for X-ray structure determination were recrystallized from ETOH by the slow evaporation of the solvent at room temperature.

### Refinement

Atom H1N3 was located from a difference Fourier maps and refined freely [N–H = 0.873 (18) Å]. The remaining H atoms were positioned geometrically [C–H = 0.93–0.97 Å] and were refined using a riding model, with *U*_iso_(H) = 1.2 or 1.5 *U*_eq_(C). A rotating group model was applied to the methyl groups. The 2-methylpent-2-ene group is disordered over two sets of sites, with a refined occupancy ratio of 0.785 (8):0.215 (8).

### Crystal data

**Table:**

C_16_H_21_N_3_O	*F*(000) = 584
*M**_r_* = 271.36	*D*_x_ = 1.109 Mg m^−^^3^
Monoclinic, *P*2_1_/*c*	Cu *K*α radiation, λ = 1.54178 Å
Hall symbol: -P 2ybc	Cell parameters from 1053 reflections
*a* = 17.5415 (8) Å	θ = 11.3–69.5°
*b* = 12.0708 (6) Å	µ = 0.56 mm^−^^1^
*c* = 7.8430 (4) Å	*T* = 296 K
β = 101.854 (3)°	Block, colourless
*V* = 1625.26 (14) Å^3^	0.90 × 0.27 × 0.17 mm
*Z* = 4	

### Data collection

**Table:**

Bruker SMART APEXII CCD area-detector diffractometer	2978 independent reflections
Radiation source: fine-focus sealed tube	2376 reflections with *I* > 2σ(*I*)
Graphite monochromator	*R*_int_ = 0.027
φ and ω scans	θ_max_ = 69.7°, θ_min_ = 5.2°
Absorption correction: multi-scan (*SADABS*; Bruker, 2009)	*h* = −16→21
*T*_min_ = 0.633, *T*_max_ = 0.912	*k* = −14→14
16548 measured reflections	*l* = −9→7

### Refinement

**Table:**

Refinement on *F*^2^	Secondary atom site location: difference Fourier map
Least-squares matrix: full	Hydrogen site location: inferred from neighbouring sites
*R*[*F*^2^ > 2σ(*F*^2^)] = 0.049	H atoms treated by a mixture of independent and constrained refinement
*wR*(*F*^2^) = 0.154	*w* = 1/[σ^2^(*F*_o_^2^) + (0.0826*P*)^2^ + 0.2272*P*] where *P* = (*F*_o_^2^ + 2*F*_c_^2^)/3
*S* = 1.03	(Δ/σ)_max_ < 0.001
2978 reflections	Δρ_max_ = 0.13 e Å^−^^3^
239 parameters	Δρ_min_ = −0.14 e Å^−^^3^
12 restraints	Extinction correction: *SHELXTL* (Sheldrick, 2008), Fc^*^=kFc[1+0.001xFc^2^λ^3^/sin(2θ)]^-1/4^
Primary atom site location: structure-invariant direct methods	Extinction coefficient: 0.0032 (6)

### Special details

**Table:**

Geometry. All s.u.'s (except the s.u. in the dihedral angle between two l.s. planes) are estimated using the full covariance matrix. The cell s.u.'s are taken into account individually in the estimation of s.u.'s in distances, angles and torsion angles; correlations between s.u.'s in cell parameters are only used when they are defined by crystal symmetry. An approximate (isotropic) treatment of cell s.u.'s is used for estimating s.u.'s involving l.s. planes.
Refinement. Refinement of F^2^ against ALL reflections. The weighted R-factor wR and goodness of fit S are based on F^2^, conventional R-factors R are based on F, with F set to zero for negative F^2^. The threshold expression of F^2^ > 2σ(F^2^) is used only for calculating R-factors(gt) etc. and is not relevant to the choice of reflections for refinement. R-factors based on F^2^ are statistically about twice as large as those based on F, and R- factors based on ALL data will be even larger.

### Fractional atomic coordinates and isotropic or equivalent isotropic displacement parameters (Å^2^)

**Table:**

	*x*	*y*	*z*	*U*_iso_*/*U*_eq_	Occ. (<1)
O1	0.30512 (6)	0.61845 (10)	0.31148 (14)	0.0640 (3)	
N1	0.06078 (9)	0.62725 (17)	0.5444 (3)	0.0897 (5)	
N2	0.40983 (7)	0.76093 (12)	0.49025 (19)	0.0656 (4)	
N3	0.33659 (7)	0.74425 (12)	0.52777 (18)	0.0602 (4)	
C1	0.09771 (12)	0.54801 (19)	0.4767 (3)	0.0905 (7)	
H1A	0.0724	0.4805	0.4511	0.109*	
C2	0.17110 (10)	0.55920 (16)	0.4418 (3)	0.0746 (5)	
H2A	0.1942	0.5007	0.3941	0.090*	
C3	0.20985 (8)	0.65855 (13)	0.47852 (19)	0.0558 (4)	
C4	0.17217 (9)	0.74139 (15)	0.5489 (2)	0.0686 (5)	
H4A	0.1959	0.8099	0.5756	0.082*	
C5	0.09864 (10)	0.72159 (19)	0.5792 (3)	0.0822 (6)	
H5A	0.0742	0.7785	0.6274	0.099*	
C6	0.28809 (8)	0.67137 (13)	0.43211 (19)	0.0534 (4)	
C7	0.45447 (9)	0.82229 (15)	0.6011 (2)	0.0645 (4)	
H7A	0.4366	0.8509	0.6957	0.077*	
C8	0.53221 (10)	0.84735 (17)	0.5801 (3)	0.0746 (5)	
H8A	0.5485	0.8169	0.4846	0.089*	
C9	0.58251 (11)	0.91090 (18)	0.6876 (3)	0.0837 (6)	
C10	0.66541 (16)	0.9344 (4)	0.6696 (6)	0.0871 (10)	0.785 (8)
H10A	0.6713	1.0139	0.6600	0.105*	0.785 (8)
H10B	0.7002	0.9109	0.7762	0.105*	0.785 (8)
C11	0.69190 (18)	0.8806 (3)	0.5187 (5)	0.0900 (11)	0.785 (8)
H11A	0.6929	0.8008	0.5341	0.108*	0.785 (8)
H11B	0.6550	0.8975	0.4118	0.108*	0.785 (8)
C12	0.77113 (18)	0.9196 (3)	0.5033 (6)	0.0828 (10)	0.785 (8)
H12A	0.7710	0.9863	0.4439	0.099*	0.785 (8)
C13	0.8396 (4)	0.8782 (5)	0.5571 (15)	0.0811 (19)	0.785 (8)
C14	0.9134 (3)	0.9352 (6)	0.5393 (11)	0.1003 (15)	0.785 (8)
H14A	0.9011	1.0045	0.4798	0.150*	0.785 (8)
H14B	0.9416	0.8891	0.4739	0.150*	0.785 (8)
H14C	0.9448	0.9486	0.6529	0.150*	0.785 (8)
C15	0.8582 (5)	0.7702 (6)	0.6537 (11)	0.162 (3)	0.785 (8)
H15A	0.8121	0.7420	0.6863	0.242*	0.785 (8)
H15B	0.8977	0.7823	0.7564	0.242*	0.785 (8)
H15C	0.8766	0.7175	0.5797	0.242*	0.785 (8)
C10A	0.6475 (6)	0.9475 (14)	0.5992 (19)	0.0871 (10)	0.215 (8)
H10C	0.6314	0.9371	0.4743	0.105*	0.215 (8)
H10D	0.6577	1.0258	0.6210	0.105*	0.215 (8)
C11A	0.7184 (8)	0.8848 (17)	0.663 (4)	0.169 (13)	0.215 (8)
H11C	0.7112	0.8104	0.6156	0.203*	0.215 (8)
H11D	0.7257	0.8787	0.7887	0.203*	0.215 (8)
C12A	0.7911 (7)	0.9321 (12)	0.620 (4)	0.123 (7)	0.215 (8)
H12B	0.7971	1.0081	0.6089	0.148*	0.215 (8)
C13A	0.8464 (13)	0.8625 (17)	0.599 (5)	0.080 (8)	0.215 (8)
C14A	0.9212 (14)	0.901 (3)	0.562 (6)	0.170 (16)	0.215 (8)
H14D	0.9258	0.9793	0.5797	0.255*	0.215 (8)
H14E	0.9232	0.8837	0.4434	0.255*	0.215 (8)
H14F	0.9633	0.8641	0.6388	0.255*	0.215 (8)
C15A	0.8294 (13)	0.7391 (13)	0.588 (4)	0.137 (8)	0.215 (8)
H15D	0.7743	0.7273	0.5742	0.206*	0.215 (8)
H15E	0.8562	0.7036	0.6925	0.206*	0.215 (8)
H15F	0.8468	0.7084	0.4895	0.206*	0.215 (8)
C16	0.56438 (16)	0.9641 (3)	0.8453 (4)	0.1287 (12)	
H16A	0.5169	0.9337	0.8683	0.193*	
H16B	0.5584	1.0424	0.8265	0.193*	
H16C	0.6061	0.9505	0.9432	0.193*	
H1N3	0.3250 (10)	0.7786 (15)	0.617 (2)	0.063 (5)*	

### Atomic displacement parameters (Å^2^)

**Table:**

	*U*^11^	*U*^22^	*U*^33^	*U*^12^	*U*^13^	*U*^23^
O1	0.0566 (6)	0.0690 (7)	0.0683 (7)	−0.0046 (5)	0.0170 (5)	−0.0065 (5)
N1	0.0508 (8)	0.1053 (13)	0.1165 (14)	−0.0154 (8)	0.0259 (8)	−0.0068 (10)
N2	0.0505 (7)	0.0773 (9)	0.0744 (8)	−0.0136 (6)	0.0255 (6)	−0.0094 (7)
N3	0.0465 (7)	0.0721 (9)	0.0658 (8)	−0.0116 (6)	0.0201 (6)	−0.0093 (6)
C1	0.0625 (10)	0.0804 (13)	0.1331 (18)	−0.0218 (10)	0.0307 (11)	−0.0046 (12)
C2	0.0580 (9)	0.0658 (10)	0.1026 (13)	−0.0102 (8)	0.0223 (9)	−0.0010 (9)
C3	0.0449 (7)	0.0622 (9)	0.0593 (8)	−0.0041 (6)	0.0088 (6)	0.0055 (7)
C4	0.0483 (8)	0.0737 (11)	0.0836 (11)	−0.0076 (7)	0.0132 (7)	−0.0115 (8)
C5	0.0489 (9)	0.0978 (14)	0.1012 (14)	−0.0048 (9)	0.0183 (9)	−0.0194 (11)
C6	0.0462 (7)	0.0559 (8)	0.0584 (8)	−0.0027 (6)	0.0113 (6)	0.0051 (6)
C7	0.0512 (8)	0.0720 (10)	0.0741 (10)	−0.0121 (7)	0.0217 (7)	−0.0097 (8)
C8	0.0554 (9)	0.0845 (12)	0.0895 (12)	−0.0165 (8)	0.0281 (8)	−0.0174 (9)
C9	0.0561 (10)	0.0823 (13)	0.1158 (15)	−0.0172 (9)	0.0251 (10)	−0.0202 (11)
C10	0.0477 (14)	0.0936 (18)	0.117 (3)	−0.0193 (15)	0.0097 (16)	−0.015 (2)
C11	0.0575 (16)	0.111 (2)	0.107 (2)	−0.0183 (15)	0.0294 (15)	−0.0033 (18)
C12	0.0540 (15)	0.0843 (19)	0.113 (2)	−0.0069 (13)	0.0249 (15)	0.0191 (18)
C13	0.078 (3)	0.077 (3)	0.093 (4)	0.007 (2)	0.026 (2)	0.018 (3)
C14	0.0549 (18)	0.110 (4)	0.140 (3)	0.004 (2)	0.030 (2)	0.001 (3)
C15	0.162 (6)	0.127 (5)	0.209 (7)	0.022 (4)	0.068 (5)	0.075 (5)
C10A	0.0477 (14)	0.0936 (18)	0.117 (3)	−0.0193 (15)	0.0097 (16)	−0.015 (2)
C11A	0.081 (9)	0.146 (15)	0.29 (3)	0.031 (9)	0.064 (14)	0.12 (2)
C12A	0.064 (7)	0.086 (8)	0.22 (2)	−0.013 (6)	0.039 (11)	0.026 (12)
C13A	0.072 (8)	0.069 (9)	0.11 (2)	0.023 (6)	0.037 (10)	0.034 (8)
C14A	0.095 (14)	0.12 (2)	0.28 (4)	0.012 (12)	0.001 (16)	−0.03 (2)
C15A	0.121 (14)	0.070 (9)	0.20 (2)	0.029 (9)	−0.008 (12)	−0.009 (10)
C16	0.0951 (17)	0.160 (3)	0.137 (2)	−0.0471 (18)	0.0378 (15)	−0.071 (2)

### Geometric parameters (Å, º)

**Table:**

O1—C6	1.2283 (18)	C12—C13	1.290 (7)
N1—C5	1.318 (3)	C12—H12A	0.9300
N1—C1	1.326 (3)	C13—C14	1.497 (6)
N2—C7	1.280 (2)	C13—C15	1.510 (7)
N2—N3	1.3903 (17)	C14—H14A	0.9600
N3—C6	1.341 (2)	C14—H14B	0.9600
N3—H1N3	0.873 (18)	C14—H14C	0.9600
C1—C2	1.377 (2)	C15—H15A	0.9600
C1—H1A	0.9300	C15—H15B	0.9600
C2—C3	1.379 (2)	C15—H15C	0.9600
C2—H2A	0.9300	C10A—C11A	1.454 (14)
C3—C4	1.375 (2)	C10A—H10C	0.9700
C3—C6	1.4985 (18)	C10A—H10D	0.9700
C4—C5	1.380 (2)	C11A—C12A	1.497 (13)
C4—H4A	0.9300	C11A—H11C	0.9700
C5—H5A	0.9300	C11A—H11D	0.9700
C7—C8	1.439 (2)	C12A—C13A	1.320 (16)
C7—H7A	0.9300	C12A—H12B	0.9300
C8—C9	1.331 (3)	C13A—C14A	1.474 (16)
C8—H8A	0.9300	C13A—C15A	1.518 (17)
C9—C16	1.485 (3)	C14A—H14D	0.9600
C9—C10	1.516 (2)	C14A—H14E	0.9600
C9—C10A	1.517 (3)	C14A—H14F	0.9600
C10—C11	1.505 (4)	C15A—H15D	0.9600
C10—H10A	0.9700	C15A—H15E	0.9600
C10—H10B	0.9700	C15A—H15F	0.9600
C11—C12	1.496 (4)	C16—H16A	0.9600
C11—H11A	0.9700	C16—H16B	0.9600
C11—H11B	0.9700	C16—H16C	0.9600
			
C5—N1—C1	116.07 (15)	C12—C13—C15	126.1 (5)
C7—N2—N3	113.78 (13)	C14—C13—C15	110.0 (6)
C6—N3—N2	118.96 (13)	C13—C14—H14A	109.5
C6—N3—H1N3	122.3 (12)	C13—C14—H14B	109.5
N2—N3—H1N3	118.6 (12)	H14A—C14—H14B	109.5
N1—C1—C2	124.24 (18)	C13—C14—H14C	109.5
N1—C1—H1A	117.9	H14A—C14—H14C	109.5
C2—C1—H1A	117.9	H14B—C14—H14C	109.5
C1—C2—C3	118.98 (18)	C13—C15—H15A	109.5
C1—C2—H2A	120.5	C13—C15—H15B	109.5
C3—C2—H2A	120.5	H15A—C15—H15B	109.5
C4—C3—C2	117.33 (14)	C13—C15—H15C	109.5
C4—C3—C6	124.20 (14)	H15A—C15—H15C	109.5
C2—C3—C6	118.40 (15)	H15B—C15—H15C	109.5
C3—C4—C5	119.13 (17)	C11A—C10A—C9	111.1 (10)
C3—C4—H4A	120.4	C11A—C10A—H10C	109.4
C5—C4—H4A	120.4	C9—C10A—H10C	109.4
N1—C5—C4	124.25 (18)	C11A—C10A—H10D	109.4
N1—C5—H5A	117.9	C9—C10A—H10D	109.4
C4—C5—H5A	117.9	H10C—C10A—H10D	108.0
O1—C6—N3	123.05 (13)	C10A—C11A—C12A	115.6 (10)
O1—C6—C3	120.88 (13)	C10A—C11A—H11C	108.4
N3—C6—C3	116.06 (13)	C12A—C11A—H11C	108.4
N2—C7—C8	120.42 (15)	C10A—C11A—H11D	108.4
N2—C7—H7A	119.8	C12A—C11A—H11D	108.4
C8—C7—H7A	119.8	H11C—C11A—H11D	107.5
C9—C8—C7	124.73 (17)	C13A—C12A—C11A	117.9 (14)
C9—C8—H8A	117.6	C13A—C12A—H12B	121.0
C7—C8—H8A	117.6	C11A—C12A—H12B	121.0
C8—C9—C16	123.28 (17)	C12A—C13A—C14A	122.2 (17)
C8—C9—C10	124.9 (2)	C12A—C13A—C15A	119.4 (16)
C16—C9—C10	111.7 (2)	C14A—C13A—C15A	118.0 (18)
C8—C9—C10A	110.0 (7)	C13A—C14A—H14D	109.5
C16—C9—C10A	124.4 (7)	C13A—C14A—H14E	109.5
C10—C9—C10A	22.7 (5)	H14D—C14A—H14E	109.5
C11—C10—C9	116.7 (3)	C13A—C14A—H14F	109.5
C11—C10—H10A	108.1	H14D—C14A—H14F	109.5
C9—C10—H10A	108.1	H14E—C14A—H14F	109.5
C11—C10—H10B	108.1	C13A—C15A—H15D	109.5
C9—C10—H10B	108.1	C13A—C15A—H15E	109.5
H10A—C10—H10B	107.3	H15D—C15A—H15E	109.5
C12—C11—C10	111.7 (3)	C13A—C15A—H15F	109.5
C12—C11—H11A	109.3	H15D—C15A—H15F	109.5
C10—C11—H11A	109.3	H15E—C15A—H15F	109.5
C12—C11—H11B	109.3	C9—C16—H16A	109.5
C10—C11—H11B	109.3	C9—C16—H16B	109.5
H11A—C11—H11B	107.9	H16A—C16—H16B	109.5
C13—C12—C11	132.1 (4)	C9—C16—H16C	109.5
C13—C12—H12A	113.9	H16A—C16—H16C	109.5
C11—C12—H12A	113.9	H16B—C16—H16C	109.5
C12—C13—C14	123.8 (6)		
			
C7—N2—N3—C6	−172.49 (15)	C7—C8—C9—C16	0.4 (4)
C5—N1—C1—C2	−0.2 (4)	C7—C8—C9—C10	177.8 (3)
N1—C1—C2—C3	0.0 (4)	C7—C8—C9—C10A	−162.8 (6)
C1—C2—C3—C4	0.0 (3)	C8—C9—C10—C11	−0.3 (6)
C1—C2—C3—C6	−177.16 (18)	C16—C9—C10—C11	177.4 (4)
C2—C3—C4—C5	0.1 (3)	C10A—C9—C10—C11	−54.2 (19)
C6—C3—C4—C5	177.14 (16)	C9—C10—C11—C12	173.7 (3)
C1—N1—C5—C4	0.3 (3)	C10—C11—C12—C13	96.6 (9)
C3—C4—C5—N1	−0.3 (3)	C11—C12—C13—C14	−175.3 (6)
N2—N3—C6—O1	−0.3 (2)	C11—C12—C13—C15	1.4 (15)
N2—N3—C6—C3	−179.34 (13)	C8—C9—C10A—C11A	−103 (2)
C4—C3—C6—O1	−149.52 (17)	C16—C9—C10A—C11A	94 (2)
C2—C3—C6—O1	27.5 (2)	C10—C9—C10A—C11A	31.7 (18)
C4—C3—C6—N3	29.5 (2)	C9—C10A—C11A—C12A	−165.1 (17)
C2—C3—C6—N3	−153.47 (16)	C10A—C11A—C12A—C13A	−149 (3)
N3—N2—C7—C8	−179.95 (16)	C11A—C12A—C13A—C14A	−178 (3)
N2—C7—C8—C9	179.4 (2)	C11A—C12A—C13A—C15A	10 (5)

### Hydrogen-bond geometry (Å, º)

**Table:**

*D*—H···*A*	*D*—H	H···*A*	*D*···*A*	*D*—H···*A*
N3—H1*N*3···O1^i^	0.873 (17)	2.052 (17)	2.9167 (18)	170.8 (16)
C4—H4*A*···O1^i^	0.93	2.53	3.251 (2)	135

Symmetry code: (i) *x*, −*y*+3/2, *z*+1/2.
